# Markov state models elucidate the stability of DNA influenced by the chiral 5S-Tg base

**DOI:** 10.1093/nar/gkac691

**Published:** 2022-08-18

**Authors:** Shu-dong Wang, Ru-bo Zhang, Leif A Eriksson

**Affiliations:** School of Chemistry and Chemical Engineering, Beijing Institute of Technology, South Street No. 5, Zhongguancun, Haidan District, 100081 Beijing, China; School of Chemistry and Chemical Engineering, Beijing Institute of Technology, South Street No. 5, Zhongguancun, Haidan District, 100081 Beijing, China; Department of Chemistry and Molecular Biology, University of Gothenburg, 405 30 Göteborg, Sweden

## Abstract

The static and dynamic structures of DNA duplexes affected by **5S-Tg** (**Tg**, Thymine glycol) epimers were studied using MD simulations and Markov State Models (MSMs) analysis. The results show that the **5S,6S-Tg** base caused little perturbation to the helix, and the base-flipping barrier was determined to be 4.4 kcal mol^−1^ through the use of enhanced sampling meta-eABF calculations, comparable to 5.4 kcal mol^−1^ of the corresponding thymine flipping. Two conformations with the different hydrogen bond structures between **5S,6R-Tg** and A19 were identified in several independent MD trajectories. The 5S,6R-Tg:O6H_O6_•••N1:A19 hydrogen bond is present in the high-energy conformation displaying a clear helical distortion, and near barrier-free **Tg** base flipping. The low-energy conformation always maintains Watson–Crick base pairing between **5S,6R-Tg** and A19, and **5S-Tg** base flipping is accompanied by a small barrier of ca. 2.0 *K*_B_*T* (*T* = 298 K). The same conformations are observed in the MSMs analysis. Moreover, the transition path and metastable structures of the damaged base flipping are for the first time verified through MSMs analysis. The data clearly show that the epimers have completely different influence on the stability of the DNA duplex, thus implying different enzymatic mechanisms for DNA repair.

## INTRODUCTION

Genetic stability and function can be significantly altered upon incorporation of mismatched or damaged nucleobases. Once a mismatched or damaged nucleobase is present in the DNA duplex, changes in the helix structure such as base flipping, which is thought to be an early event in the opening and unwinding of DNA for transcription and replication processes, often occur (1,2). Owing to the weakened intra-helical base pairing, it is proposed that the damaged or mismatched base spontaneously flips out of the DNA duplex with a certain probability, and consequently repair proteins recognize and capture the fully flipped-out base in the extra-helical conformation for further chemical processing (3). However, another mechanism postulates that the protein binds to and then slides through the duplex DNA, physically testing each base pair and induce base flipping (2,4,5). The biophysical nature of base flipping is hence still under debate, and accurate information on the static and dynamic structures of the damaged DNA and reliable thermodynamics and kinetics data on the process of damaged base flipping is therefore of high interest and importance (4,6,7).

5,6-Dihydro-5,6-dihydroxy thymine (thymine glycol, **Tg**) is the major oxidized product of thymidine under the stress of reactive oxygen species. Due to the chirality of the C5 and C6 atoms, **Tg** can exist as a mixture of the two pairs of *cis*- and *trans*-stereoisomers—the 5R *cis–trans* pair (5R,6S; 5R,6R) and 5S *cis–trans* pair (5S,6R; 5S,6S). It is estimated that 400 **Tg** molecules are formed per cell per day. Moreover, **Tg** is one of the predominant products from ionizing radiation (8–10). In γ-irradiated DNA, the 5R and 5S isomers have been reported to be formed in equal amounts (11,12). The generated chiral **Tg** may block DNA polymerase action (13), and affect the related repair enzyme process (8,14).

Studying the effect of chiral **Tg** on DNA helices, such as the most likely base flips, is a challenge for both experimental and theoretical approaches because the probability of important bases flipping out of the helices in the absence of proteins is extremely low. NMR has been applied to tackle this problem through imino proton exchange assays (15,16). However, theoretical studies showed that imino proton exchange occurs when the base pair opened by only 30°, which is still within the constraints of Watson–Crick hydrogen bonding (17,18). Hence, the fluctuation probed by NMR is related to base wobbling rather than flipping. In addition, since chiral **Tg** exists in the mixture of stereoisomers, it is experimentally difficult to distinguish which epimer is responsible for the observed base flipping phenomenon (19,20). In terms of theoretical studies, due to limitations in conformational sampling by conventional molecular dynamics (CMD) simulations, enhanced sampling methods have been applied to probe this event (21–23). However, this method likely leads to loss of critical information on key variables that may contribute to the base flipping (21). Fortunately, Markov state models (MSMs) and related models of molecular kinetics have recently received a surge of interest as they allow us to analyze the essential metastable structures, thermodynamics, and kinetics of the molecular system under investigation. Moreover, it is widely used to study slow processes of proteins and nucleic acids (24–27).

**Scheme 1.**
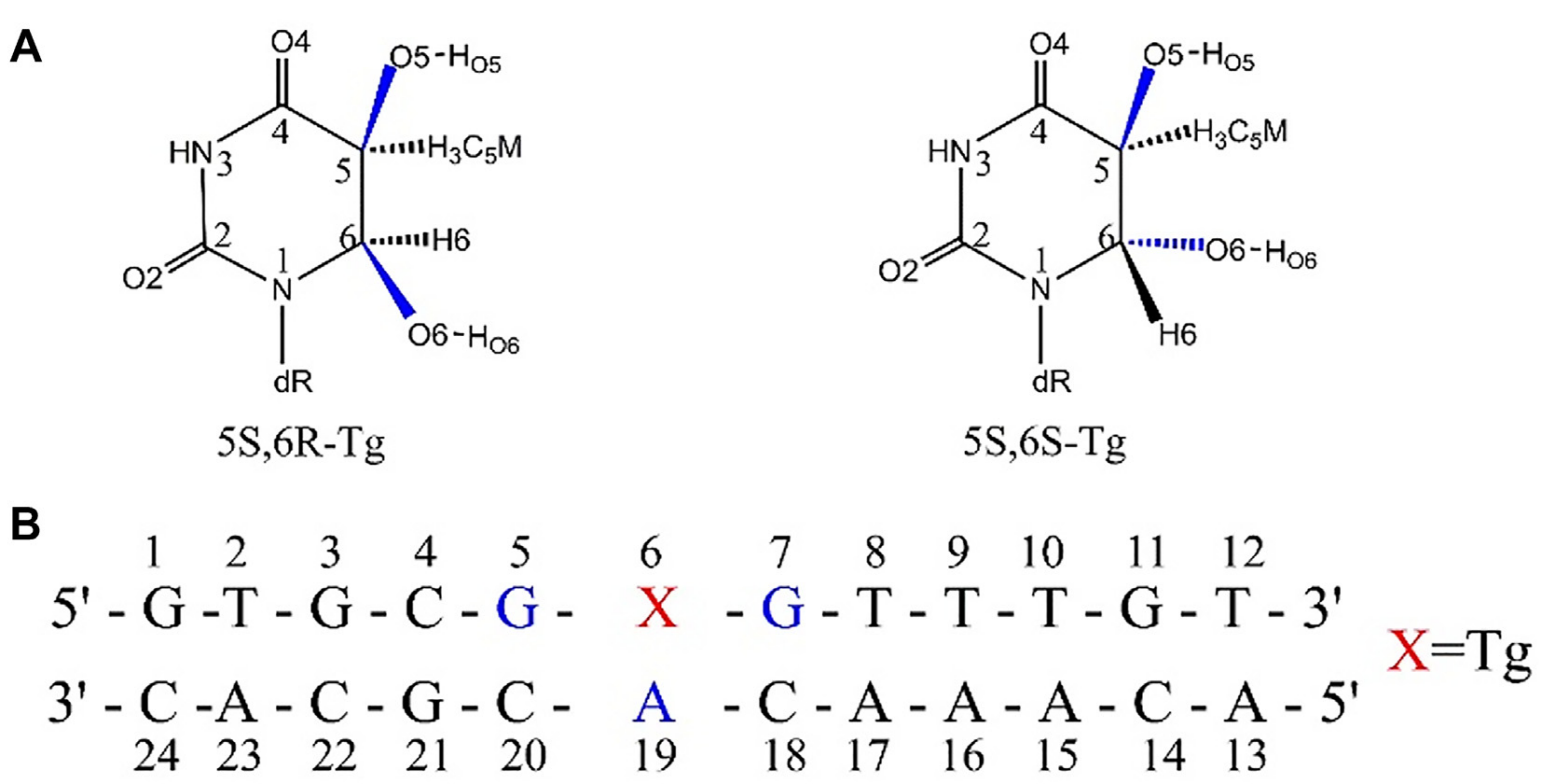
(**A**) The structures of the **5S-Tg** pair and (**B**) the sequence of the dodecamer used in the current study.

Our recent studies on **5R-Tg** unveiled for the first time that the duplex DNA with **5R,6S-Tg** was more stable than that containing **5R,6R-Tg**. Three possible conformations of the **5R,6R-Tg**-containing duplex were observed, where the high-energy conformation contributes to the **Tg** flip (28). In this work, the static and dynamic structures and energetics of the **5S,6R-** and **5S,6S-Tg** epimers (Scheme 1) were explored by all-atom molecular dynamics. The present microsecond-scale simulation results show that the **5S,6S-Tg** will remain in intra-helical conformation and forms a Watson–Crick base pair with adenine. However, **5S,6R-Tg** is observed to be extra-helical from the double helix, accompanied by a deformation of the DNA duplex. Intermolecular interactions, nucleic acid parameters and free-energies were calculated for both stable and metastable states to better understand the base interactions and conformational changes associated with base flipping. The barrier height for **5S,6S-Tg** base flipping is about 4.4 kcal mol^−1^, which is comparable to 5.4 kcal mol^−1^ for the related thymine base flipping. The **5S,6R-Tg** flipping, however, is barrier-free or needs to overcome a only 1.2 kcal mol^−1^ barrier, depending on the **Tg**:O6H_O6_ rotational structure. For a deeper insight into the variables controlling the dynamic behaviour of the ***cis*-5S,6R-Tg** base attached to the helix, multiple simulation trajectories of ***cis*-5S,6R-Tg** were used for kinetic clustering into auto-covariance modes obtained from TICA component analysis. Markov state models and flux analysis were also carried out to identify metastable states and their transition flux paths. The present conclusion strongly supports the previous hypothesis of Osman (29), suggesting that the exposed hydroxyl groups on **Tg** play an important role in the recognition by repair enzymes. Moreover, our studies for the first time reveal the kinetic process of base flipping of the damaged **5S-Tg** base out of the duplex, which provides new understanding of the stereo-selective enzymatic repair of thymine glycols.

## MATERIALS AND METHODS

Owing to the absence of **5S-Tg** containing DNA structures, the structures in our MD simulations were generated and based on the **5R,6S-Tg** containing DNA duplex structure, determined by NMR experiments (30). Subsequently, the **5S-Tg** containing DNA duplexes were separately solvated in water boxes with *ca*. 9260 TIP3P water molecules. Each system was neutralized by 0.15 M NaCl to imitate the intracellular environment. Equilibrium MD, reaching up to the microsecond time scale, were run under periodic boundary conditions using constant pressure and temperature (NPT) ensembles at 298 K and 1 atm (31,32). The CHARMM 36 force field was used throughout (33), due to it's reliability for nucleic acids (34–37), while the **5S-Tg** isomers were parameterized according to the general CHARMM procedure and fitted with the aid of the Force Field Toolkit (ffTK) (38). Lennard-Jones parameters and improper torsion parameters were taken by analogy from CHARMM’s CGenFF (39). In addition to equilibrium MD simulations, free energy profiles of the **5S-Tg** base flipping were calculated based on enhanced sampling dynamics using the recently developed extended adaptive biased force meta-dynamics (meta-eABF) approach (40,41). Through simultaneous addition of eABF biasing forces and a suitable form of the metadynamic Gaussian potentials, meta-eABF has proven particularly efficient for rapid exploration of free-energy landscapes. The algorithm possesses remarkable convergence properties over a broad range of applications including DNA system (42,43). In this study, the center-of-mass (COM) separation distance between the Tg nucleotide and A19, and the pseudodihedral angle CPDb, were considered as the collective variable (CVs) in the meta-eABF simulations, respectively (23,28). CPDb was herein defined as follows: p1 is the center-of-mass of the two flanking base pairs, p2 and p3 are the centers-of-mass of the flanking phosphate groups, respectively, and p4 is the center-of-mass of the entire six-membered ring of the flipping pyrimidine.

Markov state models (MSMs) were built using the pyEMMA 2.5 software (44). The Maximum Likelihood Estimation (MLE) algorithm was used to generate a Bayesian Markov model by estimating transition rates on microstate clusters, which are further grouped into macrostates by the PCCA + algorithm. The constructed Markov state models were validated using the Chapman-Kolmogorov test and the best generated Markov models were used to calculate the flux between metastable states. To differentiate between metastable states and to understand the conformational transitions, 10 sample conformations from each metastable state were retrieved and analysed using fluctuation correlation network and binding interaction studies. Time-lagged independent component analysis (TICA) was used to further reduce dimensionality. TICA is a powerful dimensionality reduction algorithm that extracts the most kinetically relevant linear combinations of long-lived pairwise contact distances. TICA computes the time-lagged covariance matrices *C*(τ) from a given set of mean-free input data *r*(*t*) (e.g. the long-lived pairwise distances) at time *t* with the following elements:

$$\begin{equation*}{C_{ij}}\;\left( \tau \right) = \left\langle {{r_i}\left( t \right){r_j}\left( {t + \tau } \right)} \right\rangle = \mathop \sum \limits_{t = 1}^{N - \tau } {r_i}\left( t \right){r_j}\left( {t + \tau } \right)\end{equation*}$$

where τ is the lag time and *N* is the size of the data, and:

$$\begin{equation*}C\left( \tau \right)U\; = \;C\left( 0 \right)U\Lambda \end{equation*}$$

where *U* is an eigenvector matrix consisting of time-lagged independent components (ICs) as the columns and Λ is a diagonal eigenvalue matrix. The dataset *r*(*t*) is then projected onto the TICA space that maximizes the autocorrelation of the transformed coordinates. Reduction down to the desired number of dimensions was obtained by choosing a subspace of only the first few columns of *U*,

$$\begin{equation*}{z^{\rm T}}\;\left( t \right) = {r^{\rm T}}\;\left( t \right)U\end{equation*}$$

The macrostates obtained are also referred to as metastable states, because they represent long-lived states in the dynamics of the system. In MD simulations, metastable states typically encompass whole ensembles of molecular conformations that interconvert quickly within the ensemble and slowly between ensembles. These ensembles approximately map the different basins of the free energy surface (FES), and their stationary probability π, corresponds to their Boltzmann weights. The free energy for each metastable state (Si) is computed from its stationary MSMs probability π using the relation:

$$\begin{equation*}\Delta {{G}}\left( {{s_i}} \right) = - {k_{\rm B}}T{\rm ln}\left( {\sum\limits_{j \in {S_{\rm I}}} {{\pi _j}} } \right)\end{equation*}$$

where *π*_*j*_ denotes the MSMs stationary weight of the macrostate, and *k*_B_ is the Boltzmann constant.

All MD simulations were performed using NAMD 2.14 multicore CUDA package (45), together with the Colvar module (46), while trajectories were visualized and analyzed using VMD1.9.3 (47). The DNA conformational analyses were performed with Curves+ (48).

## RESULTS AND DISCUSSION

### Molecular dynamics simulations

**Figure 1.**
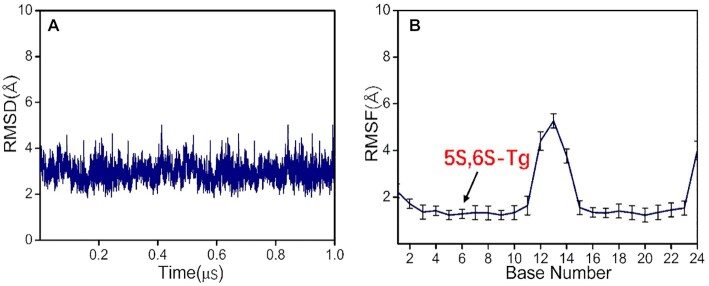
(**A**) RMSD (2.97 ± 0.41 Å), and (**B**) RMSF of each nucleotide during 1.0 μs simulation. The position of the lesion is indicated in (B).

Due to the chirality of the C6 atom, **5S-Tg** can exist as **5S,6R-** and **5S,6S-Tg** epimers. A 1.0 μs production simulation was first performed on the DNA duplex containing **5S,6S-Tg**, and root-mean-square deviation (RMSD) relative to the initial position was used to monitor the duplex structure as a measure of system stability (Figure 1A) (49). The standard deviation of the RMSD was <0.5 Å, indicating very small structural changes. Therefore, in accordance with previous studies (50), the detailed analysis was based on the last 0.1 μs trajectories. Root mean square fluctuations (RMSF) were also calculated, showing the largest fluctuations at the terminal nucleotides of the double strand (Figure 1B).

**Figure 2.**
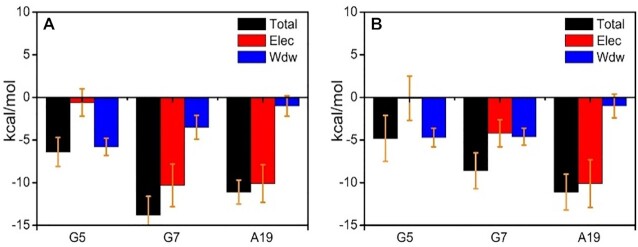
(**A**) Interaction energy decomposition of **5S,6S-Tg** with adjacent bases G5, G7 and A19; (**B**) Interaction energy decomposition of **5S,6R-Tg** with adjacent bases G5, G7 and A19 from 0.32 to 0.79 μs of the simulation.

The interaction energies of **5S,6S-Tg** with its adjacent G5, G7 and A19 bases were decomposed as shown in Figure 2A and Supplementary Table S1, and the related structure is shown in Supplementary Figure S4d. The Watson–Crick hydrogen bond energy formed between **5S,6S-Tg**/A19 is about −11.1 ± 1.4 kcal mol^−1^, which is comparable to −13.8 kcal mol^−1^ estimated at the M06-2X/6-31 + G(d,p) level (51). Note that the total interaction energy between **5S,6S-Tg** and its adjacent base G7 is as high as −13.8 ± 2.2 kcal mol^−1^ due to formation of an internucleoside hydrogen bond, in addition to π−π stacking interaction. The occupancy of this hydrogen bond during the simulation is ca. 90.3%. Contributions from G5 were also favoured, with interaction energy −6.4 ± 1.7 kcal mol^−1^. These suggest that both hydrogen-bonding and vdW effects between the bases favour stabilization of the **5S,6S-Tg** bound in double-stranded DNA. Another independent 1.0 μs replicate obtained similar results, and the corresponding RMSD and RMSF data are shown in Supplementary Figure S1.

**Figure 3.**
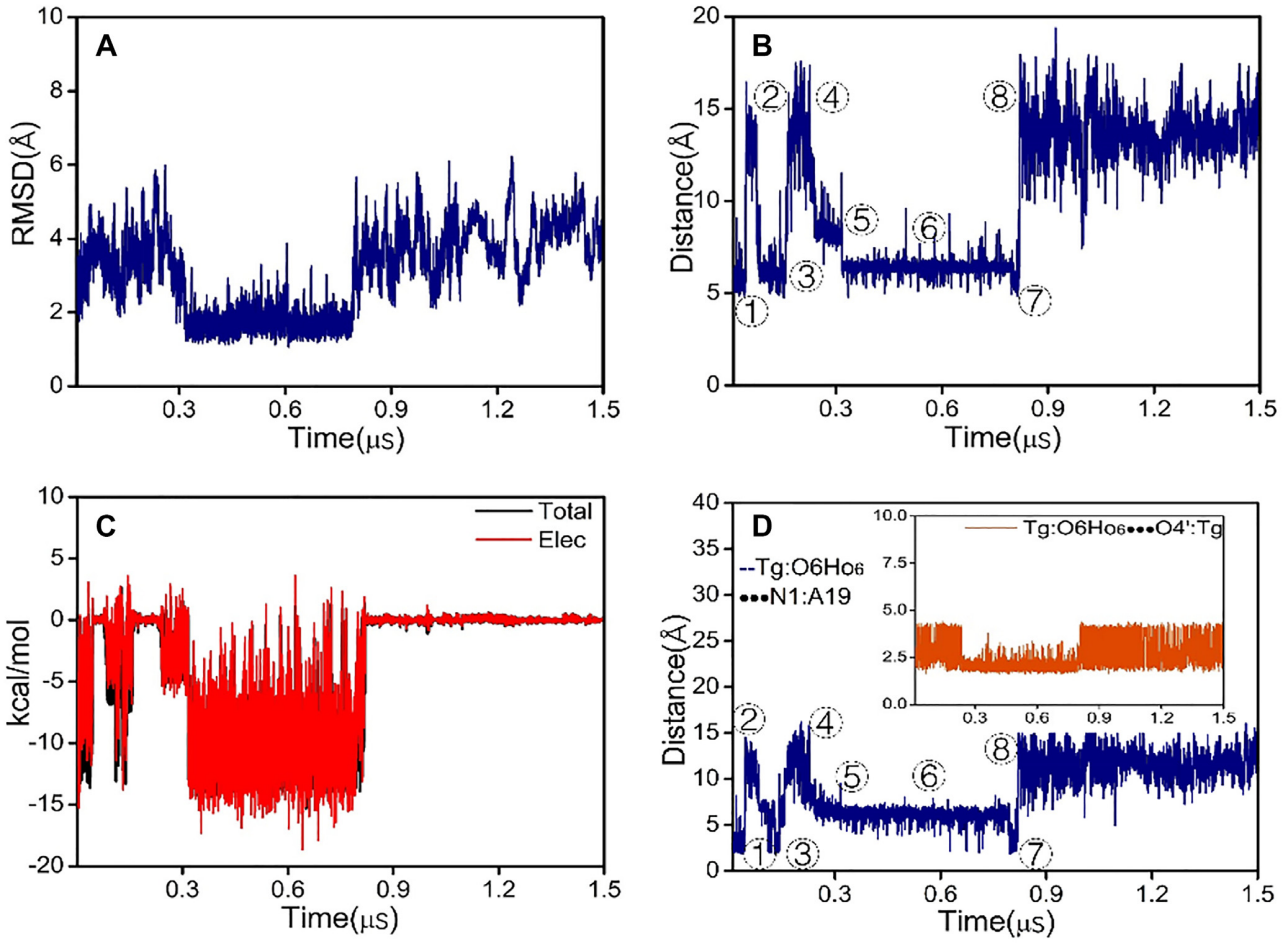
DNA with **5S,6R-Tg** during 1.5 μs simulation. (**A**) RMSD; (**B**) center of mass distance between **Tg** and A19; (**C**) interaction energy of **Tg** and A19; (**D**) The distance between **Tg**:O6H_O6_ •••N1A19(blue) and **Tg**:O6H_O6_ •••O4′A19(orange insert).

In contrast, the structural changes induced by introduction of **5S,6R-Tg** into the DNA duplex are significant. The RMSD profile during a 1.5 μs production run is displayed in Figure 3A. The center-of-mass distances between the **5S,6R-Tg** and A19 bases, and their interaction energies along the 1.5 μs trajectories are illustrated in Figure 3B and C, respectively, which show the status of **5S,6R-Tg** with respect to the helix. Several distinct regions are highlighted based on the center-of-mass distances between the **5S,6R-Tg** and A19 bases (Figure 3B). We observe that the **5S,6R-Tg** base flipping happens only in regions 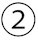, 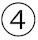 and 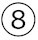 (within ca. 0.05–0.075, 0.17–0.24, 0.8–1.5 μs, respectively), and the related structure is shown in Supplementary Figure S4c. In regions 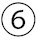 and 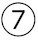, there are two different local structures associated with the **5S,6R-Tg** and A19 bases.

**Figure 4.**
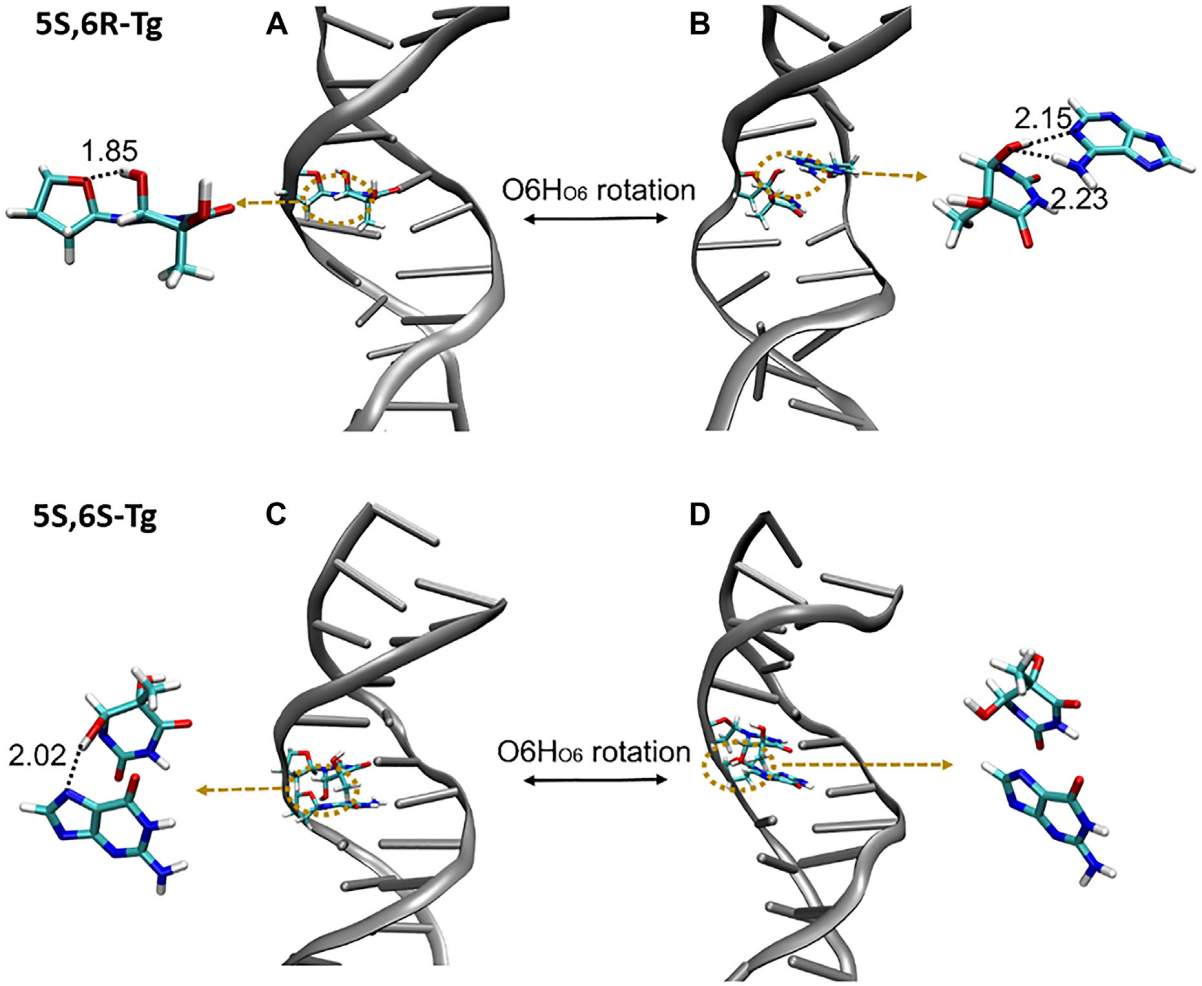
(**A**) The local **Tg**:O6Ho_6_•••O4′:**Tg** hydrogen bond observed in region 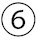
in Figure 3; (**B**) the local **Tg**:O6Ho_6_•••N1:A19 hydrogen bond observed in region 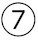
in Figure 3; (**C**) the structure of the internucleoside hydrogen bond between **Tg**:O6Ho_6_•••N7:G7 in DNA duplex containing **5S,6S-Tg**; (**D**) the structure of **5S,6S-Tg** with dangling O6H_O6_. Lengths in Å.

The classical Watson–Crick base pair between **5S,6R-Tg**/A19 is present in region 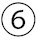, presented in Figure 4A and Supplementary Figure S4A, in which we also note an intranucleotide **Tg**:O6H_O6_•••O4′:**Tg** hydrogen bond. In region 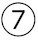, a specific short-lived **Tg**:O6H_O6_•••N1:A19 hydrogen bond is formed, along with loss of the Watson–Crick hydrogen bond between **Tg** and A19. The conformation is illustrated in Figure 4B and Supplementary Figure S4b. The two kinds of hydrogen bonds are also observed in regions 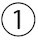 and 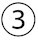, respectively. The stabilities of the hydrogen-bonded structures were also demonstrated using DFT calculations (Supplementary Figure S2). Solvent accessible surface area (SASA) often serves as a useful descriptor for the flip transition, and the plot of SASA for **Tg**/A19 vs. simulation time shown in Supplementary Figure S3 also suggests that there is a certain probability distribution of **5S-Tg** outside duplex DNA. This result is consistent with the experimental observations that the damaged or mismatched base may flip spontaneously and that the enzyme excises bases using an extrahelical base recognition mechanism (3,6,52).

According to previous studies (43,53), π-stacking interaction of the target base with its adjacent bases is one of the main factors affecting the base flipping process. Based on the locally stable structure with around 0.5 μs lifespan in region 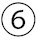 (Figure 3), we also calculated the energy decomposition of **5S,6R-Tg** with respect to interaction with the G7, G5 and A19 bases, respectively. As shown in Figure 2B and Supplementary Table S2, the hydrogen-bonding strength between **5S,6R-Tg** and A19 is comparable to that of the classical Watson–Crick base pairing seen between **5S,6S-Tg** and A19. The main difference is that the interaction of **5S,6R-Tg** with G5 and G7 is significantly weaker than the corresponding counterpart in the helix containing **5S,6S-Tg**, implying that the ability of **5S,6R-Tg** to remain aligned in the double helix becomes reduced, seen in Supplementary Figure S4A. Also, this difference is mainly due to formation of internucleosidyl hydrogen bonds between **Tg**:O6H_O6_ in **5S,6S-Tg** and N7 in G7 (Figure 4C); in **5S,6R-Tg**, **Tg**:O6H_O6_ is instead involved in an internal H-bond to **Tg**:O4′.

**Figure 5.**
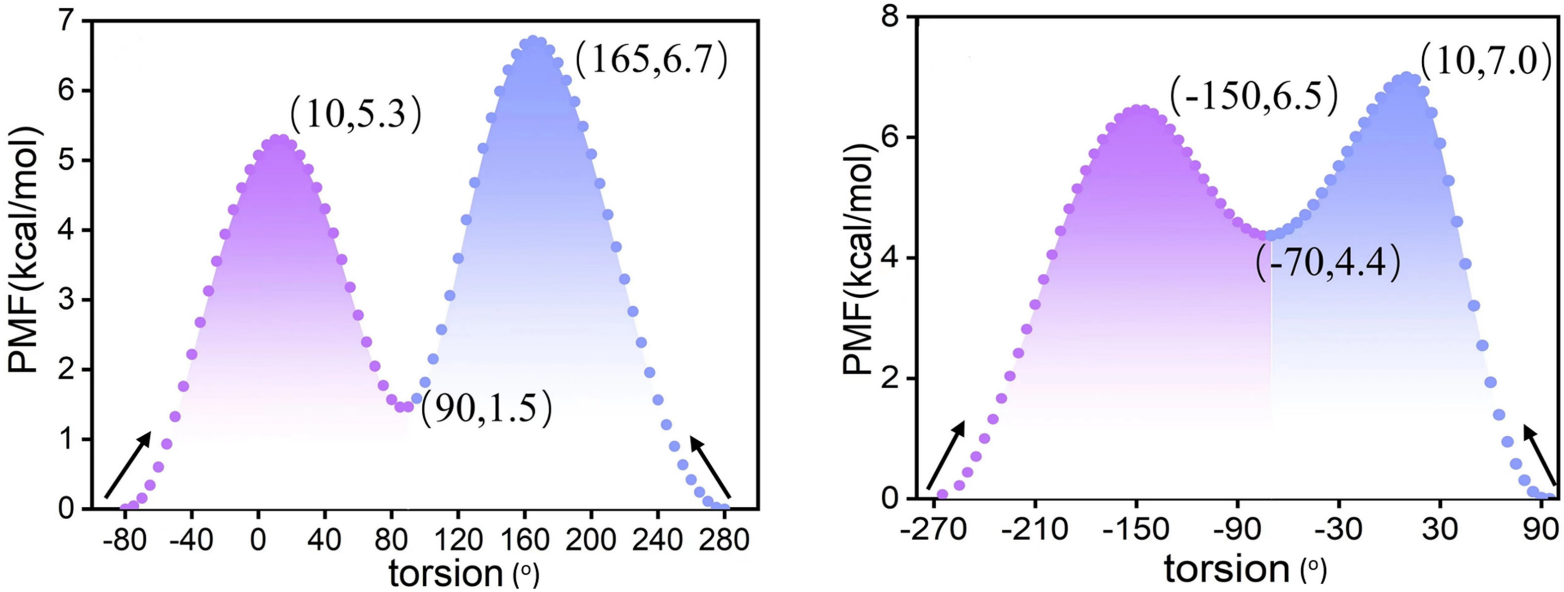
PMF profile along the torsion angle H6-C6-O6-H_O6_ reaction coordinate of (**A**) **5S,6R-Tg** containing DNA, and (**B**) **5S,6S-Tg** containing DNA. Parentheses show dihedral angle and relative energy, respectively. The purple area represents a low-energy conformation, and the blue area represents a high-energy conformation; the error is within 0.3 kcal mol^−1^ (Supplementary Figure S6).

From the helix with the local Watson–Crick structure in region 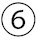 to that with the flipped **5S-Tg** base observed in region 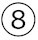, a rotational motion of O6H_O6_ in the **5S,6R-Tg** base is noted (Figure 4A and B). In order to better understand the role of this rotation, enhanced sampling dynamics along the dihedral H6-C6-O6-H_O6_ reaction coordinate was performed using meta-eABF to estimate the free energy change versus dihedral angle, seen in Figure 5A. Along the reaction coordinate, the PMF profile has minima at the dihedrals of ca. -80° (or 280°) and +90°, respectively, which correspond to the low- and high-energy conformations. The low-energy conformation includes the local **5S-Tg**:O6H_O6_•••O4′:**Tg** and Watson–Crick type **5S-Tg** •••A19 hydrogen bonds (Figure 4A), while the non-Watson–Crick **5S-Tg**:O6H_O6_•••N1:A19 hydrogen bond is found in the high-energy conformation (Figure 4B). The difference in their conformational free energies is only 1.5 kcal mol^−1^, in favour of the conformation with the Watson–Crick and local **5S-Tg:**O6H_O6_•••O4′:**Tg** hydrogen bonds. Starting from the minimum at −80°, the rotational barrier height is estimated to be 5.3 kcal mol^−1^ to reach the high-energy conformation, but only 3.8 kcal mol^−1^ for the reverse process. The transition structure is shown in Supplementary Figure S5. Therefore, the duplex with the internal **5S-Tg**:O6H_O6_••• O4′:**5S-Tg** hydrogen bond should have higher distribution within the 1.5 μs production trajectories, which corresponds to region 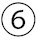 in Figure 3. We note that due to the distortion of the helix, the center-of-mass distance between **5S-Tg** and A19 is 9.4 Å in the high energy conformation, which is smaller than the corresponding distance of 10.9 Å observed in the low-energy conformation.

Meta-eABF simulations were also performed along the H6-C6-O6-H_O6_ reaction coordinate for the **5S,6S-Tg** DNA system, as shown in Figure 5B. Two minima are also here observed on the PMF curves, which are related to the **5S-Tg**:O6H_O6_ bonding status. The difference is that in the low-energy conformation near 90° (or −270°), **5S,6S-Tg**:O6H_O6_ can form a hydrogen bond with N7:G7 (Figure 4C). The high-energy conformation is less stable by 4.4 kcal mol^−1^ at about −70° dihedral angle, and has a dangling **5S,6S-Tg**:O6H_O6_ bond surrounded by solvent water molecules in the major groove (Figure 4D). The transition structure between these is also shown in Supplementary Figure S5. The rotational barrier height for **5S,6S-Tg**:O6H_O6_ is estimated to be 6.5 kcal mol^−1^, and the reverse barrier is 2.1 kcal mol^−1^. These results suggest that the high-energy conformation containing **5S,6S-Tg** should be relatively weakly occupied.

### Base flipping processes

Base flipping has come into focus recently since it is strongly relevant in some significant biological processes. Van der Vaart *et al.* found that free energy for thymine flipping is sequence dependent. The potential barrier of T flipping is ca. 10 kcal mol^−1^ (54–56). However, the choice of computational method, selection of collective variables (CV) and simulation time has been seen to influence the free-energy barrier of T base flipping. For *e.g*. the GTG fragment, the barrier ranges from 5.3 kcal mol^−1^ to 7.5 kcal mol^−1^ (28,54,57).

In order to further study this process, the PMF of **5S-Tg** base flipping for the **5S,6S-Tg** and **5S,6R-Tg** containing DNA systems were calculated with the center-of-mass distance as CV. The results are shown in Supplementary Figure S7. The barrier height for **5S,6S-Tg** base flipping is 4.4 kcal mol^−1^ (Supplementary Figure S7c), which is comparable to 5.4 kcal mol^−1^ for thymine base flipping in the intact DNA duplex. **5S,6S-Tg** base flipping is thus not considered to readily occur, which is consistent with its structural features observed in the MD trajectories. For **5S,6R-Tg**, base flipping could occur through the two above–mentioned conformations. In the high-energy conformation with **5S,6R-Tg**:O6Ho_6_•••N1:A19 hydrogen bond in the duplex, **5S,6R-Tg** base flipping is barrier free (Supplementary Figure S7b). The corresponding low-energy conformation in the **5S,6R-Tg**–containing DNA duplex, having the internal **5S,6R-Tg**:O6Ho_6_•••O4′:**5S,6R-Tg** hydrogen bond, needs to overcome a barrier of 1.2 kcal mol^−1^ to achieve the **5S,6R-Tg** base flipping (Supplementary Figure S7a). The small barrier height is close to 2.0 times the thermal energy (*k*_B_*T* is 0.60 kcal mol^−1^ at *T* = 298 K). Attack of water molecules at the weakened conformation plays a significant role for the low barrier. The center-of-mass distance is in this case 12.1 Å between **5S,6R-Tg** and A19 due to the breakdown of the hydrogen bonds, and is larger than 9.4 Å observed for the counterpart in the high-energy conformation. We can thus conclude that **5S,6R-Tg** base flipping can occur through either a barrier-free reaction starting from a high-energy conformation, or as a very low barrier reaction at the low-energy conformation. Considering the relatively high rotation barrier of O6H_O6_, it is possible that both low-barrier **5S-Tg** base flippings occur.

The **Tg** can flip through either major or minor groove pathways, but the observed events for flipping through the minor groove pathway are fewer than through the major groove (Supplementary Figure S8). For comparison, the pseudodihedral angle CPDb was used as CV for **Tg** flipping (Supplementary Figure S9). The barrier height is 2.3 kcal mol^−1^ for **5S,6R-Tg** flipping in the low energy conformation and 1.4 kcal mol^−1^ for the high energy conformation and 6.0 kcal mol^−1^ for **5S,6S-Tg** flipping from duplex DNA; close to the values 1.2, 0.0 and 4.4 kcal mol^−1^ calculated with the center-of-mass distance as reaction coordinate, respectively (Table 1). The rotational energy barrier is in general larger by ∼2 kcal mol^−1^ when using CPDb rather than distance as CVs. This slight difference may be due to insufficient sampling of the late stages of base flipping, since the flipping angle maintains roughly the same value after the flipping distance reaches a certain value. The flipping angle is thus unable to fully describe the progress along the flipping path. In comparison, the distance as a collective variable provides a better description of the late stages of the base flipping (4).

**Table 1.** Free energy barrier (kcal mol^−1^) of **5S,6R-Tg, 5S,6S-Tg** and Thymine (T) flipping from the DNA duplex using different reaction coordinates (CV’s)

Reaction coordinate	5S,6R^a^	5S,6R^b^	5S,6S	T
Distance	1.2	0	4.4	5.4
CPDb	2.3	1.4	6.6	7.1

^a^Low energy conformation.

^b^High energy conformation.

### Markov state models

The study of base flipping is very challenging for both experimental and computational methods due to the low likelihood of base flipping occurring in the stable structures of nucleic acids. Markov state models (MSMs) is an effective approach to describe kinetic landscapes of biomolecules and the transition between metastable states. To this end, Markov state models were built to identify the kinetically relevant metastable states and their transition rates (44).

In order to observe the conformational changes, we performed six independent 1.5 μs all-atom simulations of the **5S,6R-Tg** containing helix. The constructed MSMs are based on all the trajectories of these molecular dynamics simulations.

**Figure 6.**
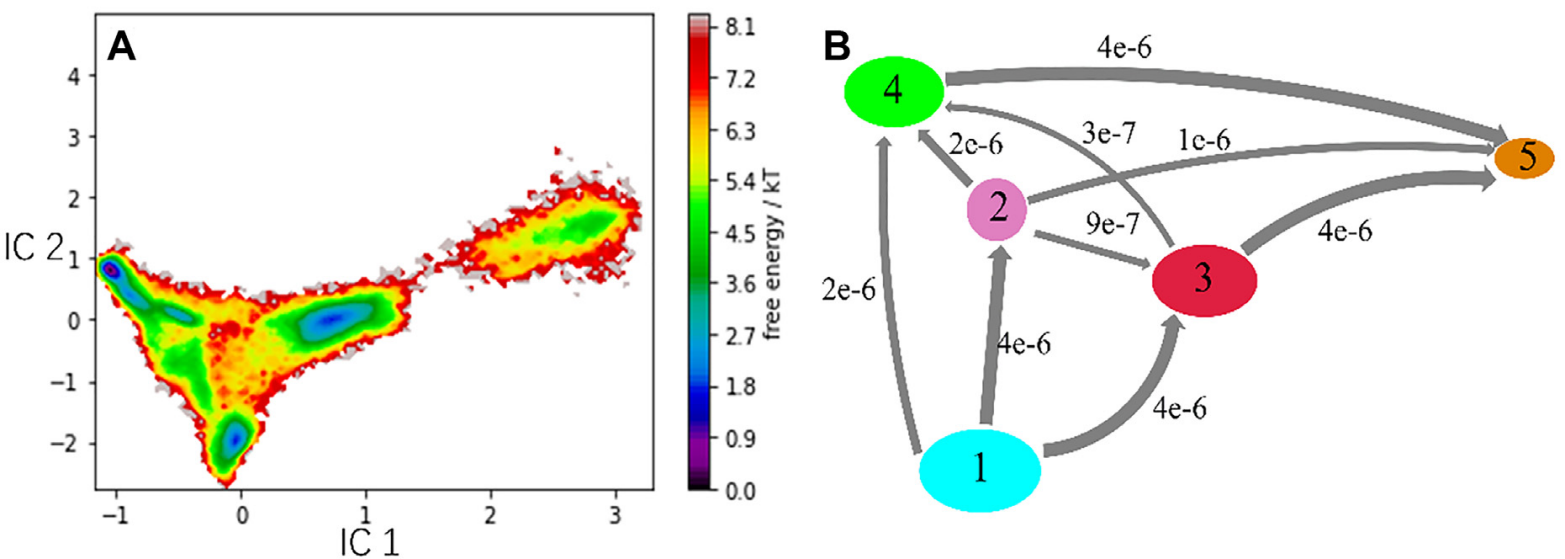
(**A**) Free energy surface projected onto the first two independent components (ICs). (**B**) Transition path flux analysis. The grey arrows between macrostates indicate the probability flux between pairs of states at equilibrium, and the arrow thickness is proportional to the flux.

The distances between all atoms of **Tg** and the complementary A19 were used as input features yielding 54 dimensions. We used a 100 ns lag time for the time-lagged independent component analysis (TICA) and reduced the feature dimensions to 30. 90% of relevant kinetic information for both flipping and intra-helical form was retained for analysis. All conformations from the MD simulations were clustered into 300 microstates by the *k-*means method. MSMs were subsequently constructed with different lag times, and τ = 100 ns was considered as a suitable lag time (Supplementary Figure S10). The 300 microstates at 100 ns lag time was grouped into 5 macrostates using PCCA+ (Perron-Cluster cluster-Analysis). The Markov models were further validated with 95% confidence level by the Chapman–Kolmogorow test (Supplementary Figure S11). The free energy surface was finally computed and projected onto the first two TICA components, seen in Figure 6A.

**Figure 7.**
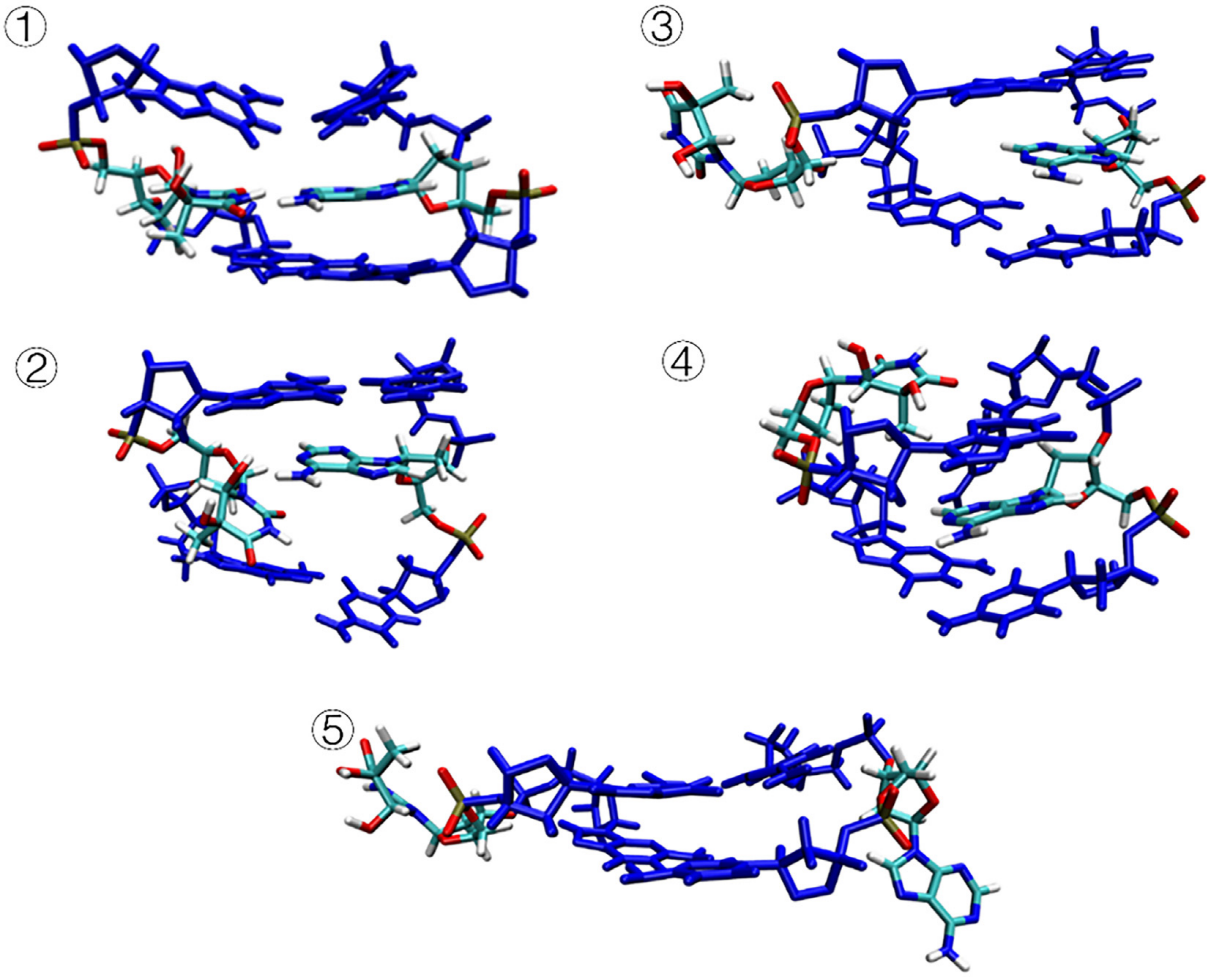
The most probable structures of each state from the MSM analysis.

The MSMs and free energy surface provide relevant information about the conformational changes of inhelix **5S,6R-Tg** towards their flipping states, with the different macrostates (Figure 7) located in the minima of Figure 6A. Macrostate 1 represents the initial closed state with Watson–Crick **5S-Tg**•••A19 hydrogen bonds (Figures 4A and 7). The structure of macrostate 2 has hydrogen bonds between **Tg**:O6H_O6_•••N1:A19. They are very similar to those observed in our MD trajectories and meta-eABF calculations for the low- and high- energy conformers of **5S,6R-Tg**, respectively. The lengths of **Tg**:O6H_O6_•••N1:A19 and A19:H61•••O6:**Tg** are ca. 2.33 and 2.31 Å, respectively, close to the values 2.15 and 2.23 Å obtained in the MD simulation, respectively (Figure 4B). From Figure 6A, we see that state 1 is clearly more stable than state 2, which is consistent with the results from the PMF and DFT calculations.

In macrostate 3, only **5S-Tg** is flipped, while A19 is still located in the double helix, and macrostate 4 corresponds to a structure in which **5S-Tg** disturbs the 5′G: C base pair, seen in Figure 7. Thereby, the Watson–Crick hydrogen bonds between **5S-Tg** and A19 disappear. Instead, **5S-Tg**:O5H_O5_•••N3:5′G and **5S-Tg**:O4•••H22:5′G hydrogen bonds are formed at the *minor* groove side. The experimentally reported interference of **Tg** on the 5′-base pair is hence confirmed by our present results (8,58). The data furthermore show that the reason why **Tg** blocks DNA polymerase is through the misplaced position of the hydrogen bond with 5′G for the **Tg** - DNA polymerase complex. MSMs analysis furthermore give macrostate 5, representing the overall flipping of **5S-Tg** and its opposing A19 base. State 3 is also observed in our meta-eABF calculations. Macrostate 5 is highly possible since solvation of A19 by water molecules entering the ‘vacant’ site once **5S-Tg** has flipped out, is inevitable. Based on Figure 6A, state 5 is less stable than state 3, which is consistent with the result from our meta-eABF calculations. DNA structural parameters of the obtained states were further analyzed using Curves+ (48). Due to the presence of Tg within the duplex, the bend angle of the DNA duplex is significantly larger that in native DNA. In addition, the Tg·A19 base-pair parameters, such as shear, stretch, stagger, buckle, prople, opening, incline and tip, are much increased compared to those for thymine in intact DNA. The major groove width of intact DNA is ∼11.4 Å (Supplementary Table S3), which in states 2 and 4 increase to 15.1 and 12.1 Å, respectively. All these changes are favourable for base flipping (59,60).

To understand the transition paths between the observed metastable states in the MSMs, flux analysis was performed. Figure 6B describes the transition paths between states 1 through 5. The flux analysis suggests that the most likely transition path between states 1 and 5 needs to go through state 3 (path 1), with a probability of ca. 37.9%. Again, a rough estimation of the free energy barrier going from state 1 to state 3 is 2.7 kcal mol^−1^, which is close to the 2.3 kcal mol^−1^ obtained with CPDb as the CV. The transition path corresponds to **5S-Tg** flipping out of the low-energy conformation on the rotational potential energy surface of **5S-Tg**:O6H_O6_. The three other possible transformation paths are 1→2→4→5 (path 2), 1→4→5 (path 3) and 1→2→5 (path 4) with probabilities 21.0%, 19.5% and 12.3%, respectively. Note that the summed probabilities of path 2, 3 and 4 are *ca*. 52.8%, showing that these transition paths can not be ignored. The transition paths 2 and 4 indicate that the **5S-Tg** base flipping must go through an intermediate state 2 with loss of the **5S-Tg**•••A19 Watson–Crick hydrogen bonds. State 2, which includes the **5S-Tg**:O6H_O6_•••N1:A19 hydrogen bond, is present as an intermediate in paths 2 and 4, and corresponds to **5S-Tg** flipping out of the high-energy conformation in the rotational potential energy surface of **5S-Tg**:O6H_O6_. The transition rate between metastable states 2 or 4 to state 5 is 1 × 10^−6^ ns^−1^ and 4 × 10^−6^ ns^−1^, respectively.

## CONCLUSIONS

Using the well-known thymine glycol as an example, microsecond-scale molecular dynamics simulations were used to address the influence of epimers on the stability of DNA supramolecular assemblies. To our knowledge, this is the first comparative study of the structures of DNA duplexes containing **5S,6R-Tg** and **5S,6S-Tg**, and the first time all-atom molecular dynamics simulations and MSMs are combined to explore flipping of the damaged nucleobase. Compared to enhanced sampling methods, MSMs provide accurate and efficient algorithms for kinetic model construction, and give reliable thermodynamic and kinetic data for the process of base flipping.

The DNA duplex containing **5S,6S-Tg** has comparable stability to the corresponding intact DNA. The epimer gives very little distortion of the DNA duplex conformation. Energy decomposition analysis shows that intermolecular hydrogen-bonding interaction contributes significantly to the binding of **5S,6S-Tg** in the duplex DNA. Two stable duplex structures containing **5S,6R-Tg** were observed in our MD studies, depending on the **5S-Tg**:O6H_O6_ rotation. A **5S-Tg**:O6H_O6_•••N1:A19 hydrogen bond is present in the high-energy conformation. In the low-energy conformation, an intranucleotide **5S-Tg**:O6H_O6_•••O4′:**5S-Tg** hydrogen bond is formed, in addition to the classical Watson–Crick hydrogen bonds between **5S,6R-Tg** and A19. The stabilities and presence of the different hydrogen bonded structures were confirmed by DFT calculations and MSMs analysis.

The activation barrier for **5S,6S-Tg** base flipping out of the duplex DNA is ca. 4.4 kcal mol^−1^. This is comparable to the 5.4 kcal mol^−1^ computed for thymine base flipping in the intact DNA, showing that **5S,6S-Tg** is stably positioned in the duplex DNA. However, the activation barrier for **5S,6R-Tg** to flip out of the double-helix DNA ranges from barrier-free to 1.2 kcal mol^−1^, depending on the local conformation. The attack of solvent water molecules plays a significant role in the breakdown of the hydrogen bonds between the bases. MSMs analysis revealed four main paths of **Tg** flipping, with the final state showing both **Tg** as well as the opposing base A19 on the complementary strand being flipped out of the helix. The present results provide detailed structural information of DNA duplexes containing **5S-Tg** epimers and new insight on how **Tg** blocks DNA polymerase. It furthermore serves as a basis for understanding the recognition of the **5S-Tg** epimers by repair enzymes.

## DATA AVAILABILITY

Simulation protocols and trajectories, optimized DFT structures, and data from MSMs analysis are provided as tarballs (.tar.gz) freely accessible at zenodo.org with DOI: 10.5281/zenodo.6563015.

## Supplementary Material

gkac691_Supplemental_File
