# Supplementary material for: Markov state models elucidate the stability of DNA influenced by the chiral 5S-Tg base
Source: Nucleic Acids Res. 2022 Aug 18;50(16):9072–82. doi: 10.1093/nar/gkac691 (PMC9458442; doi:10.1093/nar/gkac691)
Supplement: gkac691_Supplemental_File [file gkac691_supplemental_file.pdf]

Supporting Information for

Markov State Models elucidate the Stability of DNA Influenced by the Chiral 5S-Tg base

Shu-dong Wang [a], Ru-bo Zhang [a*] and, Leif A Eriksson[b*]

[a]School of Chemistry and Chemical Engineering, Beijing Institute of Technology, South Street No.5,Zhongguancun, Haidian District, 100081 Beijing, China; [b]Department of Chemistry and Molecular Biology, University of Gothenburg, 405 30 Göteborg, Sweden

[*] Corresponding authors: leif.eriksson@chem.gu.se; zhangrubo@bit.edu.cn

Table of Contents

Figure S1. RMSD and RMSF of **5S6S-Tg** DNA during 1.0 μs replica simulation…………..……...S2

Figure S2. Optimized structures of **5S,6R-Tg**/A19……………………………………………....S2

Figure S3. RMSF of **5S,6R-Tg** DNA and SASA of **5S,6R-Tg**/A19 during 1.5 μs simulation……..…S3

Table S1. Interaction energy decomposition of **5S,6S-Tg** with its adjacent G5, G7 and A19……....S3

Table S2. Interaction energy decomposition of **5S,6R-Tg** with its adjacent G5, G7 and A19……....S3

Figure S4. Overlap of different states of **5S-Tg** containing DNA with intact T containing DNA......S4

Figure S5. Transition state of the dihedral H6-C6-O6-H_{O6} rotation………………..…………..…S5

Figure S6. PMF profile along the torsion angle H6-C6-O6-HO6 reaction coordinate of **5S6R-Tg** containing DNA ………………………...…………………………………………………..…..S5

Figure S7. PMF profiles of T/Tg flipping out of the duplex with distance as the reaction coordinates……………………………………………………………………………………...S6

Figure S8. CPDb dihedral angle distribution of the 5S,6R-Tg flipping out of the duplex during the 1.5μs simulation…………………………………………………...…………………………S6

Figure S9. PMF profiles of T/Tg flipping out of the duplex with CPDb as the reaction coordinates.S7

Figure S10. Validation of the MSMs..……………………………………...……...…..……S7

Figure S11. Cross-validation of Markov state models using Chapman- Kolmogorov test…………..S8

Table S3. DNA structure parameters calculated with Curves+……………………...……...……S9

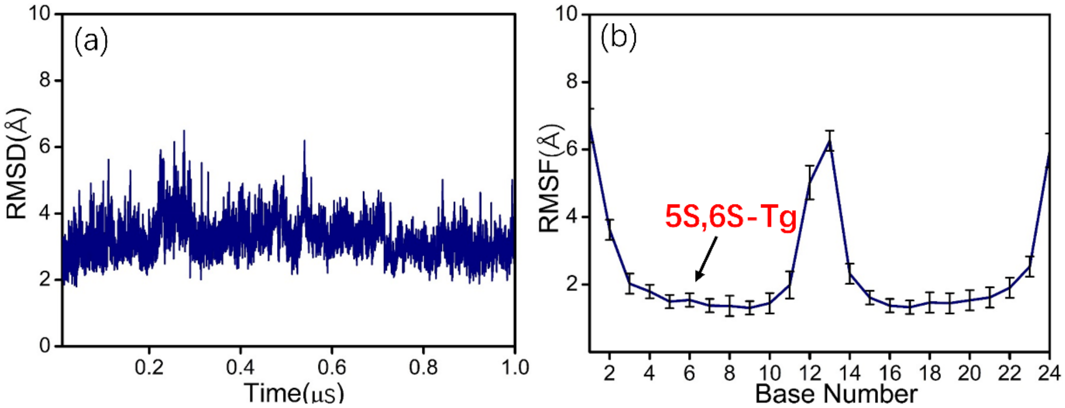

Figure S1. (a) RMSD (2.97±0.41 Å) and **(b)** RMSF of each nucleotides of 5**S,6S-Tg** DNA during 1.0 μs replica simulation.

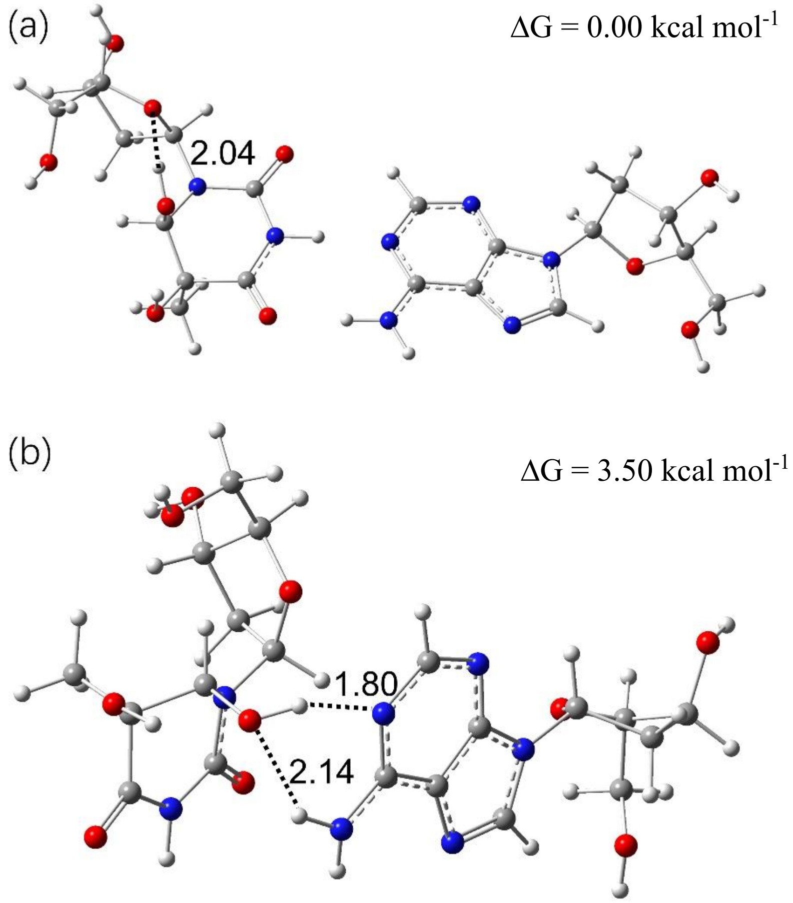

Figure S2. (a) Optimized structures of the low energy conformation with internal **5S,6R-Tg**:O6H$_{O6}$•••O4′:**5S,6R-Tg** hydrogen bond at the M06-2X/6-31G(d,p) level; **(b)** Optimized structures of the high energy conformation with local **5S,6R-Tg**:O6H$_{O6}$•••N1:A19 hydrogen bonds at the M06-2X/6-31G(d,p) level.

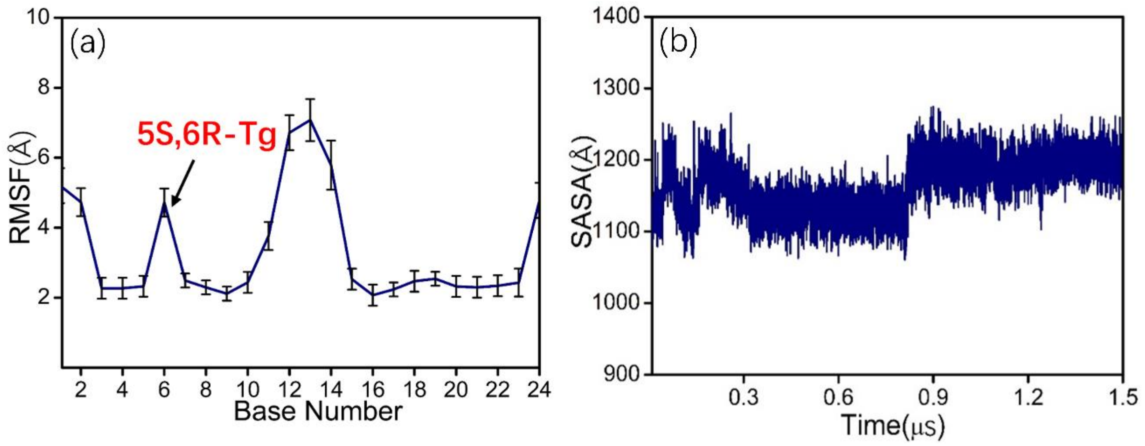

Figure S3. (a) The RMSF of each nucleotides of **5S,6R-Tg** DNA and **(b)** SASA (Å) of **5S,6R-Tg**/A19 during 1.5 μs simulation.

Table S1. Interaction energy decomposition (kcal mol^{-1}) of **5S,6S-Tg** with its adjacent bases G5, G7 and A19.

Interaction energy	**5S6S-Tg**		
	G5	G7	A19
Elec	-0.6±1.6	-10.3±2.5	-10.1±2.2
Vdw	-5.8±1.0	-3.5±1.4	-1.0±1.2
Total	-6.4±1.7	-13.8±2.2	-11.1±1.4

Table S2. Interaction energy decomposition (kcal mol^{-1}) of **5S,6R-Tg** with its adjacent bases G5, G7 and A19.

Interaction energy	**5S,6R-Tg**			**5S,6R-Tg**		
	G5	G7	A19	G5	G7	A19
Elec	-0.1±2.6	-4.2±1.6	-10.1±2.8	-2.3±0.6	-4.1±0.8	-6.8±2.3
Vdw	-4.7±1.1	-4.4±1.0	-1.0±1.4	0.3±0.8	-4.0±2.3	-2.0±1.0
Total	-4.8±2.7	-8.6±2.1	-11.1±2.1[a]	-2.0±0.9	-8.1±2.0	-8.8±2.1[b]

[a] Interaction energy decomposition during time span 0.32 to 0.79 μs, corresponding to the low-energy conformation with a **5S,6R-Tg**:O6H_{O6}•••O4′:**5S,6R-Tg** hydrogen bond.
[b]Interaction energy decomposition during the time span 0.79 to 0.80 μs, corresponding to the high-energy conformation with a **5S,6R-T**g:O6H_{O6}•••N1:A19 hydrogen bond.

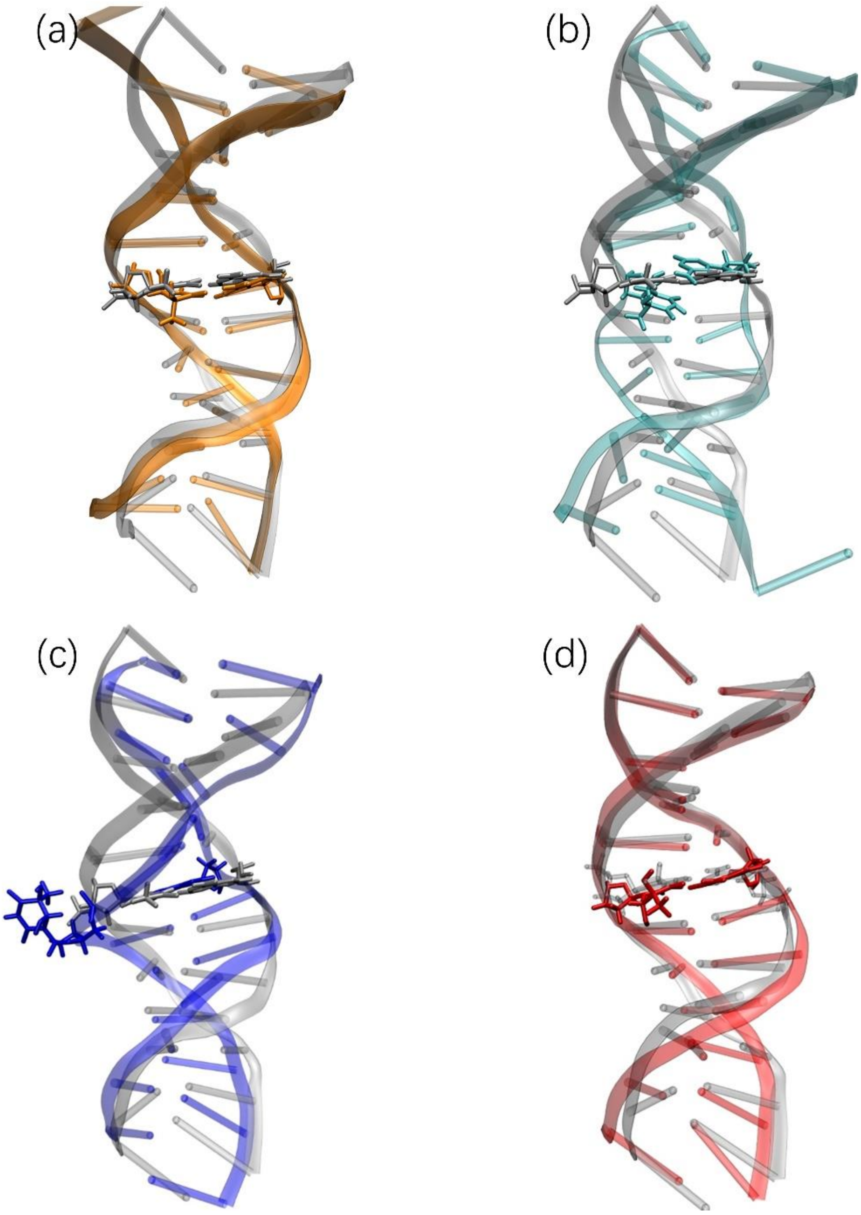

Figure S4 Overlap of different states of **5S-Tg** containing DNA with intact T containing DNA (in silver). **(a) 5S,6R-Tg** intra-helical state (in orange)**; (b) 5S,6R-Tg**:O6H$_{O6}$•••N1:A19 hydrogen bond state (in cyan)**; (c) 5S,6R-Tg** flipping state (in blue); **(d) 5S,6S-Tg** containing DNA (in red).

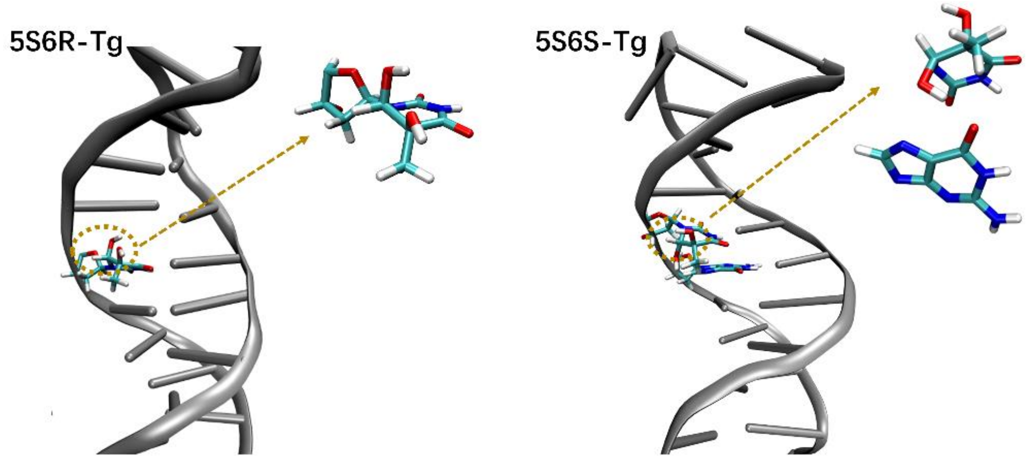

Figure S5. The transition structures for the dihedral H6-C6-O6-H_{O6} rotation.

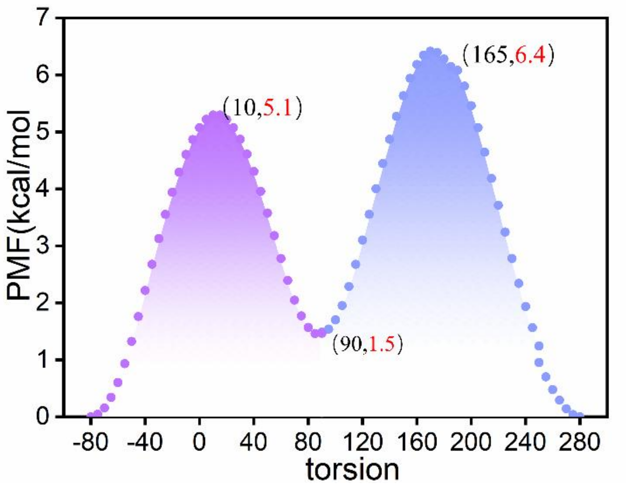

Figure S6. PMF profile along the torsion angle H6-C6-O6-H_{O6} reaction coordinate of **5S6R-Tg** containing DNA for the second simulation. (The purple area represents the low-energy conformation and the blue area represents the high-energy conformation)

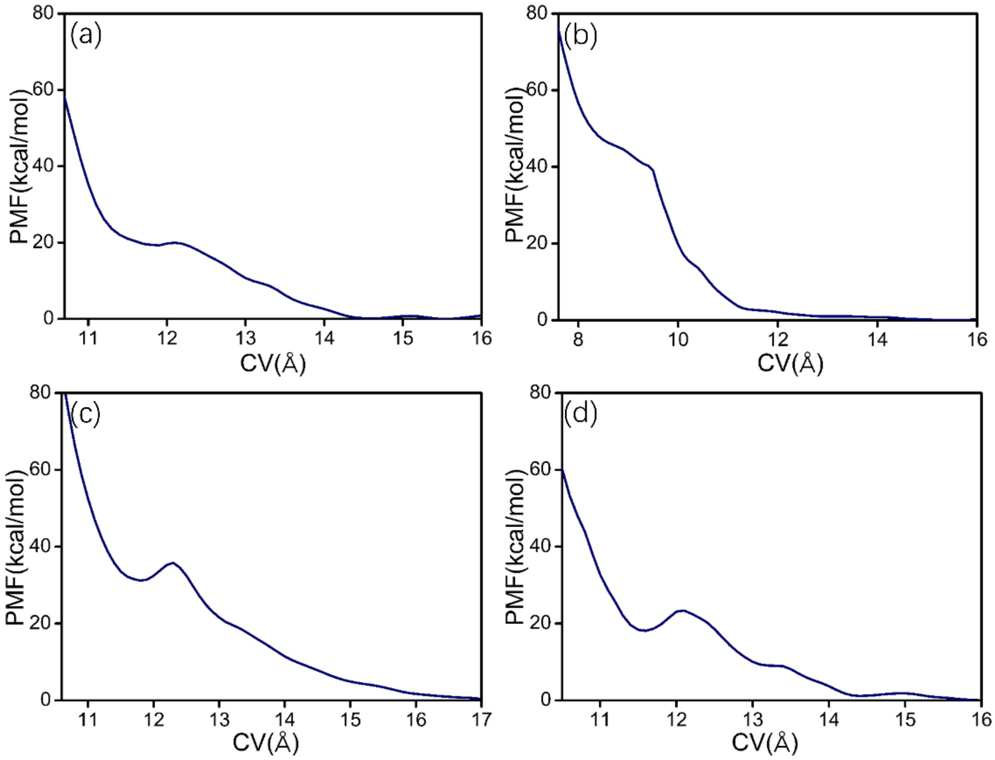

Figure S7. PMF profiles of **T/Tg** flipping out of the duplex with distance as the reaction coordinate. **(a) 5S,6R-Tg** flipping in low energy conformation, barrier 1.2 kcal mol^{-1}; **(b) 5S,6R-Tg** flipping in high energy conformation, barrier-free; **(c) 5S,6S-Tg** flipping from duplex DNA, barrier 4.4 kcal mol^{-1}; **(d) T** flipping in intact DNA, barrier 5.4 kcal mol^{-1} .

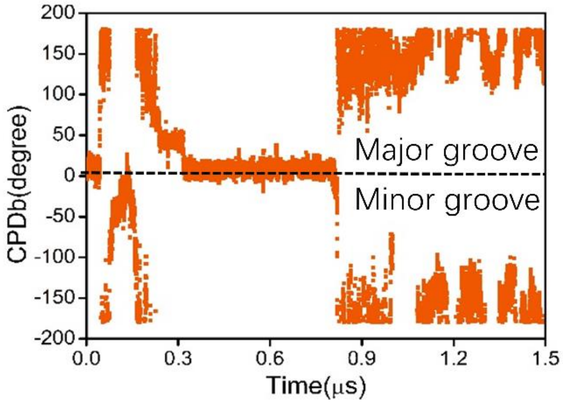

Figure S8. CPDb dihedral angle distribution for **5S,6R-Tg** flipping out of the duplex.

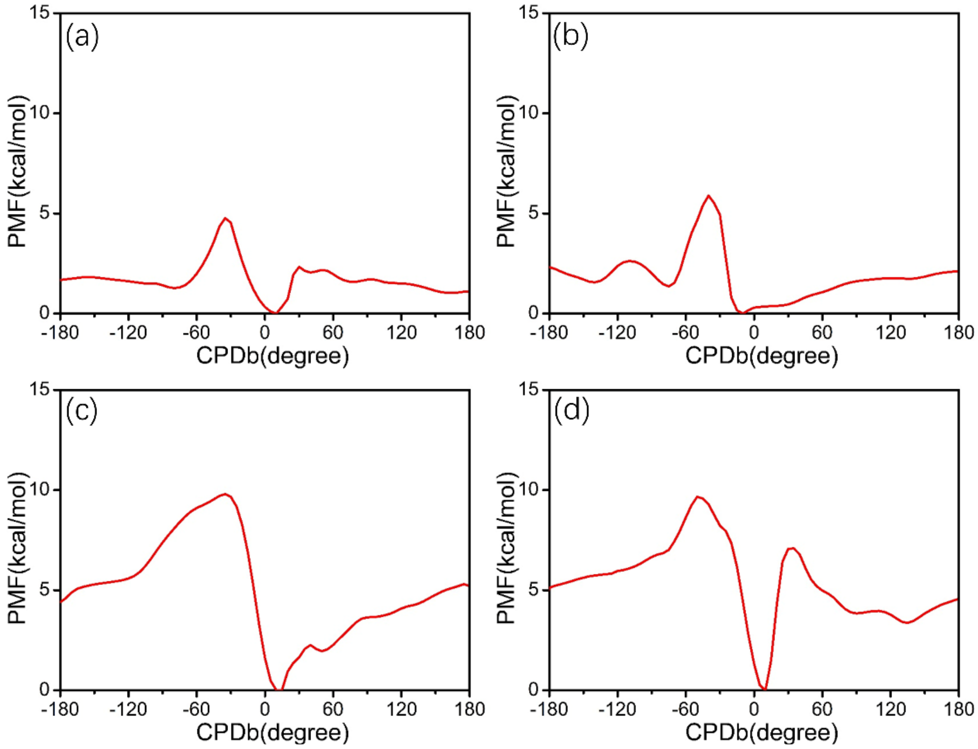

Figure S9. PMF profiles of **T/Tg** flipping out of the duplex with pseudo-dihedral angle CPDb as the reaction coordinates. **(a) 5S,6R-Tg** flipping in low energy conformation, barrier 2.3 kcal mol^{-1}; **(b) 5S,6RT-Tg** flipping in high energy conformation, barrier 1.4 kcal mol^{-1}; **(c) 5S,6S-Tg** flipping form duplex DNA, barrier 6.0 kcal mol^{-1}; **(d) T** flipping in intact DNA, barrier 7.1 kcal mol^{-1}.

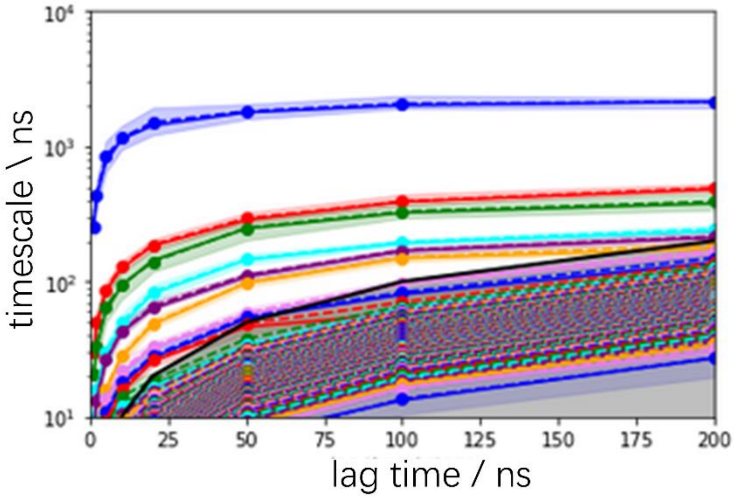

Figure S10. Validation of the MSMs. The solid lines correspond to Implied time scales of maximum likelihood, while the means are plotted as dashed lines, and the 95% confidence intervals of the means are depicted as shaded regions. The implied time scales converged at lag time of 100 ns.

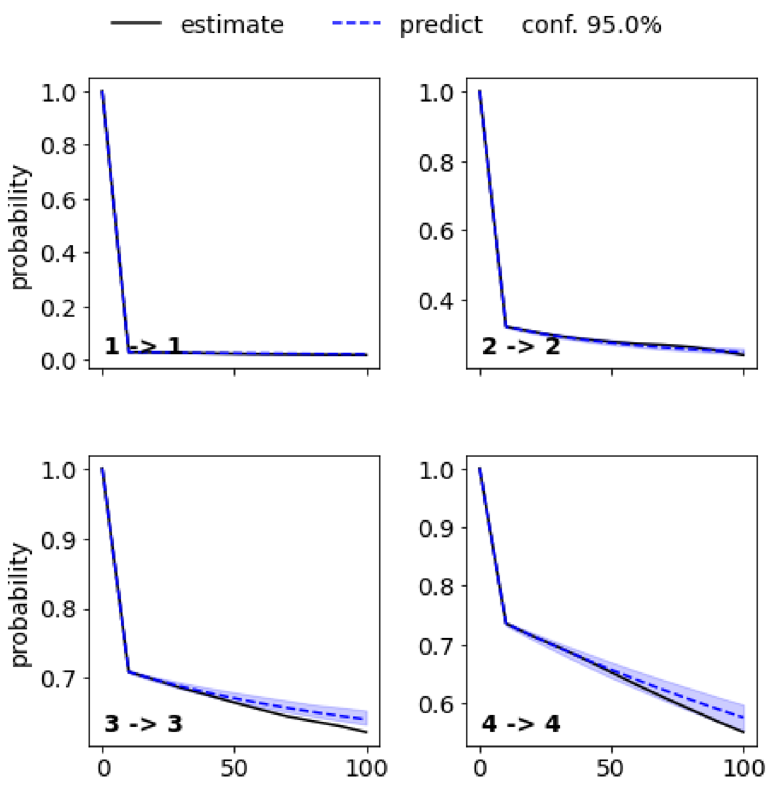

Figure S11. Cross-validation of Markov state models using Chapman- Kolmogorov test at a lag time 100ns.

Table S3. DNA structure parameters[a] calculated with Curves+. DNA-thy is thymine in intact DNA; State 1 – 5 are the five metastable states from the MSMs.

	Xdisp	Ydisp	Inclin	Tip	bend				
DNA-thy	0.76	0.89	11.5	2.1	10.6				
State1	0.21	0.61	16.4	9.2	52.0				
State2	1.79	0.35	-3	0.7	34.7				
State3	-5.05	3.3	41.2	41.5	52.5				
State4	-2.99	-1	2.2	8.5	33.3				
State5	-2.09	0.09	-29.1	-111.5	31.8				
	Shear	Stretch	Stagger	Buckle	Prople	Opening			
DNA-thy	0.16	-0.16	0.03	3.1	-11.1	-0.2			
State1	0.42	0.01	0.4	-29	-14.3	11.5			
State2	1.36	-2.64	4.11	-33	-9.5	16.1			
State3	-9.27	-0.58	-14.61	114.7	52.4	-81.7			
State4	-5.28	-4.82	-5.66	4.5	-23.1	-31.3			
State5	12.04	-23.34	15.62	-41	-100.6	18.8			
	Shift	Slide	Rise	Tilt	Roll	Twist	H-rise	H-Twi	
DNA-thy	-0.37	2	3.74	0.4	-4.1	43	4	42.6	
State1	-0.72	0.57	2.76	1.9	3.9	33.8	2.82	35	
State2	-1.64	-1.1	4.36	7	12.4	-7.2	4.5	-7.2	
State3	1.25	-3.08	7.66	-62.5	-6	78.9	4.98	85.2	
State4	2.26	0.96	6.77	-8.1	0.8	61.7	6.49	62.3	
State5	0.36	-0.16	-0.24	144.6	7.9	51.5	0.41	136.2	
	α	β	γ	δ	ε	ζ	χ	Pha	Amp
DNA-thy	-61.2	-175.7	44.3	142.1	-90.3	178.4	-93.1	155.8	41.5
State1	-51.7	-164	37.5	143.7	-170.5	-114.4	-95.9	149.4	34.4
State2	-53.3	178.9	38.7	81.9	-99.6	-142.6	-155.5	20.3	46.3
State3	59.5	161	70.7	81.5	178.6	146.9	-165	10.4	39.1
State4	-177.1	-170.2	54.1	83.1	-116.5	-113.8	-162.8	10.6	43.8
State5	32.9	-166	48.1	84.9	169.2	177.8	-173.6	-3.2	50.8
	Min-W[a]	Min-D[b]	Maj-W[a]	Maj-D[b]					
DNA-thy	7.9	5	11.4	4.8					
State1	8.9	3.5	9.6	6.9					
State2	9.9	-0.5	15.1	4.3					
State3	6.8	6.9	10.7	4.4					
State4	8	-1.4	12.1	5.4					
State5	3.7	5.3	8	4.1					

[a] See Curves+ for definition of structural parameters, http://curvesplus.bsc.es/.
